# Ethnic differences in cardiovascular risk: examining differential exposure and susceptibility to risk factors

**DOI:** 10.1186/s12916-022-02337-w

**Published:** 2022-04-27

**Authors:** Frederick K. Ho, Stuart R. Gray, Paul Welsh, Jason M. R. Gill, Naveed Sattar, Jill P. Pell, Carlos Celis-Morales

**Affiliations:** 1 Institute of Health and Wellbeing, University of Glasgow, 1 Lilybank Gardens, Glasgow, G12 8RZ UK; 2 Institute of Cardiovascular & Medical Sciences, Glasgow, UK

**Keywords:** Ethnicity, Cardiovascular, Obesity, Deprivation

## Abstract

**Background:**

Ethnic differences in cardiovascular disease (CVD) risk have been known for decades, but a systematic exploration of how exposure and susceptibility to risk factors may contribute is lacking. This study aimed to investigate the potential impact of differential exposure and susceptibility between South Asian, Black, and White individuals.

**Methods:**

This is a population-based prospective cohort study of UK Biobank participants with a median follow-up of 11.3 years. The association between ethnic group and CVD risk was studied. Additional risk factors were then adjusted to examine mediations. Moderation analysis was conducted to identify whether risk factors had a stronger association in the ethnic minority groups. Population attributable fractions were also calculated to quantify the relative contributions of risk factors for each ethnic group.

**Results:**

When adjusted for only age and sex, there was a higher risk of CVD among South Asian (*n*=8815; HR [95% CI] 1.69 [1.59–1.79]) and Black (*n*=7526; HR [95% CI] 1.12 [1.03–1.22]) compared with White participants (*n*=434,809). The excess risk of Black participants was completely attenuated following adjustment for deprivation. Compared with White participants, the associations of BMI, triglycerides, and HbA1c with CVD were stronger in South Asians. Adiposity was attributable to the highest proportion of CVD regardless of ethnicity. Smoking had the second largest contribution to CVD among White and Black participants, and HbA1c among South Asian participants.

**Conclusions:**

Adiposity is an important risk factor for CVD regardless of ethnicity. Ethnic inequalities in CVD incidence may be best tackled by targeting interventions according to ethnic differences in risk profiles.

**Supplementary Information:**

The online version contains supplementary material available at 10.1186/s12916-022-02337-w.

## Background

Ethnic inequalities in health have been known for decades. Studies have consistently shown that ethnic minority groups in Western high-income countries were at a higher risk of mortality [1, 2] and a wide range of morbidities, including cardiovascular disease (CVD) [3], the main contributor to all-cause mortality.

The excess risk experienced by ethnic minority groups is largely attributable to historical and current socioeconomic inequalities and cultural factors rather than genetic differences [4]. A conceptual framework has suggested that health inequalities can be decomposed into two components: differential exposure and differential susceptibility [5]. The former postulates that ethnic minority groups have a higher prevalence of risk factors for disease, e.g. South Asians had a higher prevalence of diabetes [6], a known risk factor for CVD, while the latter indicates that a risk factor has a stronger effect on the ethnic minority group, e.g. higher BMI associated with COVID-19 mortality more strongly in non-White ethnic groups [7]. In reality, both may apply [5].

In the UK, an electronic record linkage study of over one million patients showed that South Asians were at 67% and 29% higher risk of myocardial infarction and ischaemic stroke respectively, while Black people were at 51% lower risk of myocardial infarction and 24% higher risk of ischaemic stroke [8]. The study also explored the differential exposure hypothesis, in terms of body mass index (BMI), systolic blood pressure (SBP), and smoking. However, they noted only negligible differences in the associations when these were adjusted, in part due to the selective assessment and reporting of these factors in primary care settings [8]. We have previously shown that differential susceptibility between ethnic groups also applies in relation to diabetes and CVD [6].

To date, there has been no systematic exploration of the extent to which differential exposure and differential susceptibility explain ethnic inequalities in CVD across a wide range of risk factors. Similarly, there have been no comparisons of the relative contributions of risk factors to CVD between ethnic groups [3]. This study, therefore, aimed to investigate the existence and impact of differential exposure and susceptibility to cardiovascular risk factors between South Asian, Black, and White participants in UK Biobank. In this study, we included incident heart failure (HF) in our definition of CVD as its prevalence has increased while that of myocardial infarction (MI) has declined. In this way, we believe the present study allows a more holistic analysis of CVD risks by ethnicity.

## Methods

### Study design and participants

UK Biobank is a prospective cohort study. Between 2007 and 2010, UK Biobank recruited over 500,000 participants from the general population. Participants attended one of 22 assessment centres across England, Scotland, and Wales where they completed a self-administered, touch-screen questionnaire and face-to-face interview, and trained staff took a series of phenotypic measurement and biological samples. Participants who withdrew from the UK Biobank, including those who have left the UK (*n*=1099), were excluded from this study.

### Exposure

Participants’ ethnicity was self-reported as baseline in a touch-screen questionnaire and was categorised as White, Black, and South Asian.

### Outcomes

Outcomes were ascertained through individual-level record linkage of the UK Biobank cohort to routine administrative databases. Date and cause of death were obtained from death certificates held by the National Health Service Information Centre (England and Wales) and the National Health Service Central Register Scotland (Scotland). Date and cause of hospital admissions were obtained through record linkage to Health Episode Statistics (England and Wales) and Scottish Morbidity Records (Scotland). Detailed information about the linkage procedures can be found at http://content.digital.nhs.uk/services. At the time of analysis, hospital admission data were available up to 31 March 2017 and mortality data up to 30 April 2020. We defined CVD deaths using the European Society of Cardiology SCORE definition [9] (ICD-10 [International Classification of Diseases, 10th revision] codes I10-25, I47-50, I60-69, and I70-73) and incident CVD cases as CVD deaths or hospital admissions for myocardial infarction (I20, I21, I25), heart failure (I50), or stroke (I60-64).

### Covariates

We developed models based on modifiable risk factors, including lifestyle, anthropometric parameters, and biomarkers. Television viewing time, dietary intake of food items, smoking status, and alcohol consumption were self-reported. Townsend area deprivation index was derived from the postcode of residence using aggregated data on unemployment, car and home ownership, and household overcrowding [10]. Systolic blood pressure (SBP) was measured by a trained nurse. Physical activity was self-reported using the validated International Physical Activity Questionnaire [11]. Grip strength was measured to the nearest 0.1 kg using a Jamar J00105 hydraulic hand dynamometer and the mean value from both hands used in the analyses. Height was measured to the nearest centimetre, using a Seca 202 stadiometer, and body weight to the nearest 0.1 kg, using a Tanita BC-418 body composition analyser. Body mass index (BMI) was calculated as weight/height^2^ and the World Health Organisation’s criteria were used to classify BMI into underweight (<18.5 kg/m^2^), normal weight (18.5 to 24.9 kg/m^2^), overweight (25 to 29.9 kg/m^2^), and obese (≥30.0 kg/m^2^). Central obesity was defined as waist-hip ratio (WHR) >0.85 for women and >0.90 for men. Biomarkers were measured at a dedicated central laboratory between 2014 and 2017. Our analyses included low-density lipoprotein cholesterol (LDL-c), triglycerides, glycated haemoglobin (HbA1c), cystatin C, and gamma-glutamyltransferase (GGT) as potential mediators. LDL-c and triglycerides are related to CVD and lifestyle factors such as smoking and obesity; HbA1c is a marker for diabetes and related to obesity; cystatin C is a marker of kidney function and related to diet, smoking, and body weight; and GGT is a marker of liver function and related to alcohol drinking and fatty liver disease. These measures were externally validated with stringent quality control [12]. High-density lipoprotein (HDL) cholesterol and C-reactive protein, two important CVD risk markers, were not included because those were not likely to be causal factors of CVD [13, 14].

### Statistical analyses

Four analyses were conducted. Firstly, Cox proportional hazard models were used to analyse the association between risk factors and CVD for each ethnic group, with the results reported as hazard ratios (HRs) and 95% confidence intervals (CIs). Risk factors were grouped as deprivation, lifestyle (physical activity, TV viewing, diet, smoking, alcohol drinking), adiposity (BMI and WHR), physical factors (grip strength and SBP), and serum-based biomarkers (LDL-c, triglycerides, HbA1c, cystatin C, GGT). All risk factors were adjusted for age at baseline assessment and sex (model 1), lifestyle risk factors additionally for deprivation (model 2), adiposity additionally for lifestyle factors (model 3), and physical factors and biomarkers additionally for adiposity (model 4). We chose these models so that we only adjusted for potential confounders, and upstream causes, not for downstream mediators on causal pathways. For example, because the factors in models 2–4 are likely mediators between deprivation and CVD, they were not adjusted to avoid overadjustment. Continuous variables were standardised to their SDs for comparison between risk factors.

Secondly, we explored if any of the risk factors could explain the association between ethnic group and CVD risk. A baseline Cox model (model 1, adjusted for age and sex only) was used to assess the total association between ethnicity and CVD. Groups of risk factors as described above were adjusted for sequentially. The HR for ethnic group was then used to examine whether, and to what extent, the associations between ethnic group and CVD were attenuated. Percentage risk reductions (%RD) after adjusting for these factors were calculated as $\% RD=\frac{HR_{M_1}-{HR}_{M_i}}{HR_{M_1}-1}$ to illustrate the magnitude of mediation [15]. This method is used instead of the counterfactual-based methods because it is hard to disentangle the interrelationship among risk factors in this study. This mediation analysis serves as an exploration of the relative importance of mediators, rather than an attempt to quantify the proportion mediated.

Thirdly, moderation analysis was conducted to quantify whether the effect estimate for a risk factor was significantly stronger or weaker in an ethnic minority group compared to White participants. The associations between risk factors and CVD were studied in each of the ethnic group. Then, the interaction terms between risk factors and ethnicity were included in Cox models and can be interpreted as the ratio of hazard ratios (HR_Black_:HR_White_ and HR_South Asian_:HR_White_). In a sensitivity analysis, the associations of SBP, LDL-c, triglycerides, and HbA1c were also studied with the adjusted of relevant diagnosis and medications (anti-hypertensive and cholesterol-lowering medications) as those could be confounders.

Lastly, the population attributable fractions of risk factors were calculated for each of the three ethnic groups in stratified analyses. These indicate the relative contributions of risk factors to CVD cases or death within each ethnic group, taking account of both the prevalence and hazard ratios of the risk factor [16, 17]. Prevalence was estimated from the included participants. Continuous variables (grip strength and serum biomarkers) were categorised by quintiles because assuming linear association between the risk factor and CVD in PAF analysis would imply all individuals could be modified to the lowest level of that risk factor which is unrealistic. The prevalence for these was calculated using the quintile associated with the highest risk, i.e. the 1st quintile for grip strength and the 5th quintile for serum biomarkers. The remaining quintiles (2nd–5th for grip strength; 1st–4th for other biomarkers) were combined and used as the reference group in estimating HRs.

To avoid inflated type I error due to multiple testing, all *P*-values presented were adjusted using Holm’s procedure and a corrected *P*-value <0.05 is considered statistically significant [18]. Proportional hazard assumptions were checked using Schoenfeld residuals, which revealed no significant violation (all *P*-values 0.05). Non-cardiovascular mortality was considered a competing event, and follow-up was censored at death [19]. All analyses were conducted using R version 4.0.2 with packages *survival*, and *AF*.

## Results

Of the 502,493 UK Biobank participants, 11,882 were excluded because they were not recorded as White, South Asian, or Black; 38,900 because they had prior or prevalent CVD at baseline; and 561 because they had missing sociodemographic data, resulting in a study population of 451,150. Of these, 434,809 (96.4%) were White, 8815 (2.0%) South Asian, and 7526 (1.7%) Black. Compared with White participants, South Asians had lower alcohol intake and higher fruit/vegetable intake and were less likely to currently smoke, but they also performed less physical activity and had higher WHR and HbA1c (Table 1). Black participants had the highest grip strength, were the most socioeconomically deprived, watched the most TV, and had the highest red meat intake and BMI.

**Table 1:** Study participant characteristics by ethnic group

	White ***n***=434,809	South Asian ***n***=8815	Black ***n***=7526
Mean (SD) age, years	56.34 (8.03)	52.65 (8.29)	51.59 (7.95)
Sex, n (%)			
Female	244,174 (56.2)	4281 (48.6)	4342 (57.7)
Male	190,635 (43.8)	4534 (51.4)	3184 (42.3)
Mean (SD) deprivation index	−1.50 (2.96)	0.30 (3.15)	2.62 (3.45)
Mean (SD) physical activity, MET-min/week	2678.77 (2507.71)	2266.76 (2322.17)	2568.82 (2519.60)
Mean (SD) TV viewing, hr/day	2.76 (1.56)	2.48 (1.56)	3.00 (1.89)
Mean (SD) fruit/vegetable intake, portion/day	4.08 (2.34)	5.05 (3.81)	4.50 (3.45)
Mean (SD) red meat intake, portion/day	2.10 (1.41)	1.47 (1.68)	2.55 (2.28)
Processed meat intake twice/day or more, n (%)	135,214 (31.1)	1653 (19.1)	1706 (23.1)
Never had oily fish, n (%)	46571 (10.8)	2544 (29.9)	395 (5.4)
Smoking, n (%)			
Never	239,235 (55.2)	6823 (78.3)	5256 (70.4)
Previous	149,470 (34.5)	1052 (12.1)	1286 (17.2)
Current	44,667 (10.3)	841 (9.6)	927 (12.4)
Mean (SD) alcohol intake, units/week	16.80 (18.89)	5.60 (12.46)	6.83 (12.61)
Mean (SD) BMI, kg/m^2^	27.26 (4.72)	27.06 (4.37)	29.41 (5.32)
BMI categories, n (%)			
Underweight	146,554 (33.8)	2864 (32.9)	1428 (19.3)
Normal	2303 (0.5)	59 (0.7)	10 (0.1)
Overweight	183,844 (42.5)	3969 (45.7)	3079 (41.6)
Obese	100,363 (23.2)	1801 (20.7)	2890 (39.0)
Waist-hip ratio	0.87 (0.09)	0.90 (0.08)	0.87 (0.08)
Central obesity, n (%)	203,420 (46.9)	5491 (63.1)	3655 (49.3)
Mean (SD) hand grip strength, kg	30.70 (11.00)	26.82 (10.51)	31.65 (11.39)
Mean (SD) SBP, mmHg	137.91 (18.65)	134.62 (18.38)	137.55 (18.56)
Mean (SD) LDL-c, mmol/L	3.62 (0.85)	3.42 (0.83)	3.28 (0.83)
Mean (SD) triglycerides, mmol/L	1.74 (1.03)	1.98 (1.22)	1.22 (0.75)
Mean (SD) HbA1c, mmol/mol	35.65 (6.15)	40.04 (10.09)	39.05 (9.69)
Mean (SD) cystatin C, mg/L	0.90 (0.16)	0.94 (0.21)	0.85 (0.20)
Mean (SD) GGT, U/L	36.53 (42.69)	36.46 (40.47)	41.58 (41.53)
Prevalent diabetes at baseline assessment, n (%)	15358 (3.5)	1142 (13.0)	693 (9.2)
Anti-hypertensive medications, n (%)	74,089 (17.0)	1868 (21.2)	2116 (28.1)
Cholesterol-lowering medications, n (%)	54,833 (12.6)	1818 (20.6)	1001 (13.3)

*SD* standard deviation, *MET* metabolic equivalent of tasks, *BMI* body mass index, *LDL-c* low-density lipoprotein cholesterol, *HbA1c* glycated haemoglobin, *GGT* gamma-glutamyl transferase

Over a median of 11.27 (IQR 10.41–12.01) years of follow-up, there were 42,062 incident CVD cases, of which 4797 were fatal. Overall, the crude CVD incidence rates were 86.43 per 10,000 person-years. Crude CVD rates were 86.2, 116.2, and 67.7 per 10,000 person-years among White, South Asian, and Black participants respectively. When adjusted for only age and sex, there was a higher risk of CVD cases among South Asian and Black participants than White participants (Table 2). In mediation analysis, the excess risk of Black participants was completely attenuated following adjustment of deprivation. That of South Asian participants was modestly attenuated in each step of additional mediators.

**Table 2:** Associations* between ethnic group and cardiovascular disease following adjustment for covariates

	South Asian			Black		
	HR (95% CI)	***P***	%RD	HR (95% CI)	***P***	%RD
Age and sex only	**1.69 (1.59–1.79)**	**<0.0001**	-	1.12 (1.03–1.22)	0.01	-
+ Deprivation index	**1.55 (1.45–1.64)**	**<0.0001**	20.3	0.90 (0.82–0.98)	0.01	183.3
+ Lifestyle factors	**1.65 (1.54–1.76)**	**<0.0001**	5.8	0.98 (0.89–1.07)	0.63	116.7
+ Adiposity	**1.58 (1.48–1.69)**	**<0.0001**	15.9	0.94 (0.85–1.03)	0.18	150.0
+ Physical factors	**1.42 (1.32–1.52)**	**<0.0001**	39.1	0.92 (0.84–1.01)	0.08	166.7
+ Serum-based biomarkers	**1.35 (1.25–1.45)**	**<0.0001**	49.3	0.94 (0.84–1.04)	0.23	150.0
All	**1.22 (1.13–1.32)**	**<0.0001**	68.1	0.91 (0.82–1.02)	0.10	166.7

*Referent to White participants

Lifestyle factors: physical activity level, television viewing, dietary intake of fruit and vegetable, red meat, processed meat, oily fish, smoking status, and alcohol drinking; adiposity: body mass index and waist-hip ratio; physical factors: grip strength and systolic blood pressure; serum-based biomarkers: low-density lipoprotein cholesterol, glycated haemoglobin, cystatin C, and gamma-glutamyl transferase

*CVD* cardiovascular disease, *HR* hazard ratio, *%RD* percentage risk difference

Associations between risk factors and CVD by ethnic group are shown in Table 3. Among White participants, all selected factors, except LDL-c, were associated with CVD cases. In South Asian participants, deprivation, low physical activity, TV viewing, adiposity, low grip strength, and high SBP, triglycerides, HbA1c, cystatin C, and GGT were associated with CVD. The same risk factors were significantly associated with CVD cases among Black participants, apart from physical activity, TV viewing, grip strength, triglycerides, and GGT. Smoking was significant among Black participants but not South Asian. After being adjusted for cholesterol-lowering medications, the association between LDL-c and CVD was significant among White and South Asians (Additional file 1: Table S1). The associations of SBP, triglycerides, and HbA1c were consistent when relevant diagnosis and medications were adjusted. There was no evidence for violation of proportional hazard assumption (all *P*s > 0.05).

**Table 3:** Associations between risk factors and cardiovascular disease by ethnic group

	White		South Asian		Black	
	HR (95% CI)	***P***	HR (95% CI)	***P***	HR (95% CI)	***P***
Deprivation index^a^	**1.17 (1.16–1.19)**	**< 0.0001**	**1.10 (1.04–1.16)**	**0.003**	**1.16 (1.08–1.26)**	**0.0002**
Physical activity level^b^	0.99 (0.98–1.00)	0.09	**0.87 (0.80–0.95)**	**0.04**	0.92 (0.83–1.03)	1.00
TV viewing^b^	**1.15 (1.14–1.16)**	**< 0.0001**	**1.11 (1.04–1.17)**	**0.02**	1.07 (1.00–1.15)	0.97
Dietary intake^b^						
Fruit and vegetable	**0.93 (0.92–0.94)**	**< 0.0001**	0.95 (0.91–1.00)	0.57	0.99 (0.92–1.05)	1.00
Red meat	**1.05 (1.04–1.06)**	**< 0.0001**	1.03 (0.98–1.08)	1.00	1.00 (0.94–1.06)	1.00
Processed meat	**1.11 (1.09–1.14)**	**< 0.0001**	1.05 (0.90–1.23)	1.00	1.23 (1.00–1.50)	0.72
Low oily fish	**1.25 (1.21–1.29)**	**< 0.0001**	1.11 (0.98–1.27)	1.00	1.16 (0.79–1.69)	1.00
Smoking status^b^						
Never	1 (Reference)		1 (Reference)		1 (Reference)	
Former	**1.20 (1.18–1.23)**	**< 0.0001**	0.93 (0.78–1.11)	1.00	**1.50 (1.22–1.86)**	**0.003**
Current	**1.94 (1.89–2.00)**	**< 0.0001**	1.16 (0.95–1.41)	1.00	**1.63 (1.27–2.10)**	**0.003**
Alcohol drinking^b^	**1.02 (1.01–1.03)**	**0.0007**	0.98 (0.89–1.07)	1.00	1.06 (0.94–1.20)	1.00
Adiposity^c^						
BMI	**1.22 (1.21–1.23)**	**< 0.0001**	**1.38 (1.29–1.47)**	**< 0.0001**	**1.25 (1.15–1.35)**	**< 0.0001**
Waist-hip ratio	**1.31 (1.29–1.33)**	**< 0.0001**	**1.43 (1.31–1.57)**	**< 0.0001**	**1.39 (1.23–1.58)**	**< 0.0001**
Physical factors^d^						
Grip strength	**0.84 (0.82–0.85)**	**< 0.0001**	**0.85 (0.77–0.94)**	**0.005**	1.00 (0.88–1.13)	1.00
Systolic blood pressure	**1.14 (1.13–1.16)**	**< 0.0001**	**1.18 (1.10–1.26)**	**< 0.0001**	**1.15 (1.04–1.26)**	**0.03**
Serum-based biomarkers^d^						
LDL-c	1.01 (1.00–1.02)	0.06	1.00 (0.93–1.07)	0.98	0.94 (0.85–1.04)	1.00
Triglycerides	**1.05 (1.04–1.06)**	**<0.0001**	**1.10 (1.04–1.17)**	**0.003**	1.08 (0.96–1.22)	0.88
HbA1c	**1.12 (1.11–1.13)**	**< 0.0001**	**1.18 (1.14–1.23)**	**< 0.0001**	**1.12 (1.05–1.19)**	**0.007**
Cystatin C	**1.19 (1.18–1.21)**	**< 0.0001**	**1.18 (1.11–1.26)**	**< 0.0001**	**1.35 (1.25–1.47)**	**< 0.0001**
GGT	**1.09 (1.08–1.10)**	**< 0.0001**	**1.13 (1.06–1.20)**	**0.001**	1.05 (0.96–1.14)	1.00

*HR* hazard ratio, *CI* confidence interval, *CVD* cardiovascular disease, *BMI* body mass index, *LDL-c* low-density lipoprotein cholesterol, *HbA1c* glycated haemoglobin, *GGT* gamma-glutamyl transferase

^a^Model 1: adjusted for age and sex only

^b^Model 2: additionally adjusted for deprivation

^c^Model 3: additionally adjusted for lifestyle factors

^d^Model 4: additionally adjusted for adiposity markers

All *P*-values were adjusted for multiple testing using Holm’s procedure

Moderation analysis findings are shown in Table 4. Compared to White participants, the associations of BMI, triglycerides, and HbA1c with CVD were stronger in South Asians and the association between cystatin C and CVD was stronger in Black participants.

**Table 4:** Ratio of associations between risk factors and cardiovascular disease among South Asian and Black participants relative to White

	South Asian		Black	
	HR ratio (95% CI)	***P***	HR ratio (95% CI)	***P***
Deprivation index^a^	0.95 (0.89, 1.00)	0.13	0.98 (0.91, 1.06)	0.69
Physical activity level^b^	**0.88 (0.81, 0.97)**	**0.01**	0.90 (0.80, 1.01)	0.07
TV viewing^b^	0.95 (0.89, 1.01)	0.12	0.93 (0.86, 1.00)	0.12
Dietary intake^b^				
Fruit and vegetable	1.00 (0.95, 1.05)	0.96	1.08 (1.01, 1.15)	0.06
Red meat	0.98 (0.92, 1.03)	0.41	0.94 (0.88, 1.00)	0.14
Processed meat	0.96 (0.82, 1.14)	1.00	0.96 (0.78, 1.19)	1.00
Low oily fish	1.02 (0.89, 1.17)	1.00	0.88 (0.58, 1.33)	1.00
Smoking status^b^				
Never	1 (Reference)		1 (Reference)	
Former	0.80 (0.67, 0.96)	0.053	1.11 (0.89, 1.39)	0.34
Current	**0.62 (0.51, 0.77)**	**<0.0001**	0.76 (0.58, 0.98)	0.07
Alcohol drinking^b^	0.96 (0.88, 1.05)	0.36	0.90 (0.80, 1.02)	0.23
Adiposity^c^				
BMI	**1.16 (1.08, 1.24)**	**<0.0001**	1.08 (1.00, 1.17)	0.06
Waist-hip ratio	1.08 (1.01, 1.16)	0.07	1.00 (0.90, 1.11)	0.95
Physiological factors^d^				
Grip strength	1.04 (0.96, 1.12)	0.70	0.98 (0.89, 1.10)	0.77
Systolic blood pressure	0.99 (0.93, 1.07)	1.00	1.01 (0.91, 1.12)	1.00
Serum-based biomarkers^d^				
LDL-c	1.01 (0.94, 1.08)	0.78	0.95 (0.86, 1.06)	0.72
Triglycerides	**1.08 (1.02, 1.14)**	**0.01**	1.03 (0.91–1.17)	0.68
HbA1c	**1.05 (1.01, 1.09)**	**0.04**	1.03 (0.96, 1.09)	0.42
Cystatin C	1.00 (0.94, 1.06)	0.91	**1.13 (1.04, 1.23)**	**0.01**
GGT	1.04 (0.97, 1.10)	0.37	0.94 (0.85, 1.03)	0.37

HR ratios are the interaction terms HR_South Asian_:HR_White_ and HR_Black_:HR_White_

*CVD* cardiovascular disease, *HR* hazard ratio, *CI* confidence interval, *BMI* body mass index, *LDL-c* low-density lipoprotein cholesterol, *HbA1c* glycated haemoglobin, *GGT* gamma-glutamyl transferase

^a^Model 1: adjusted for age and sex only

^b^Model 2: additionally adjusted for deprivation

^c^Model 3: additionally adjusted for lifestyle factors

^d^Model 4: additionally adjusted for adiposity markers

**Fig. 1:**
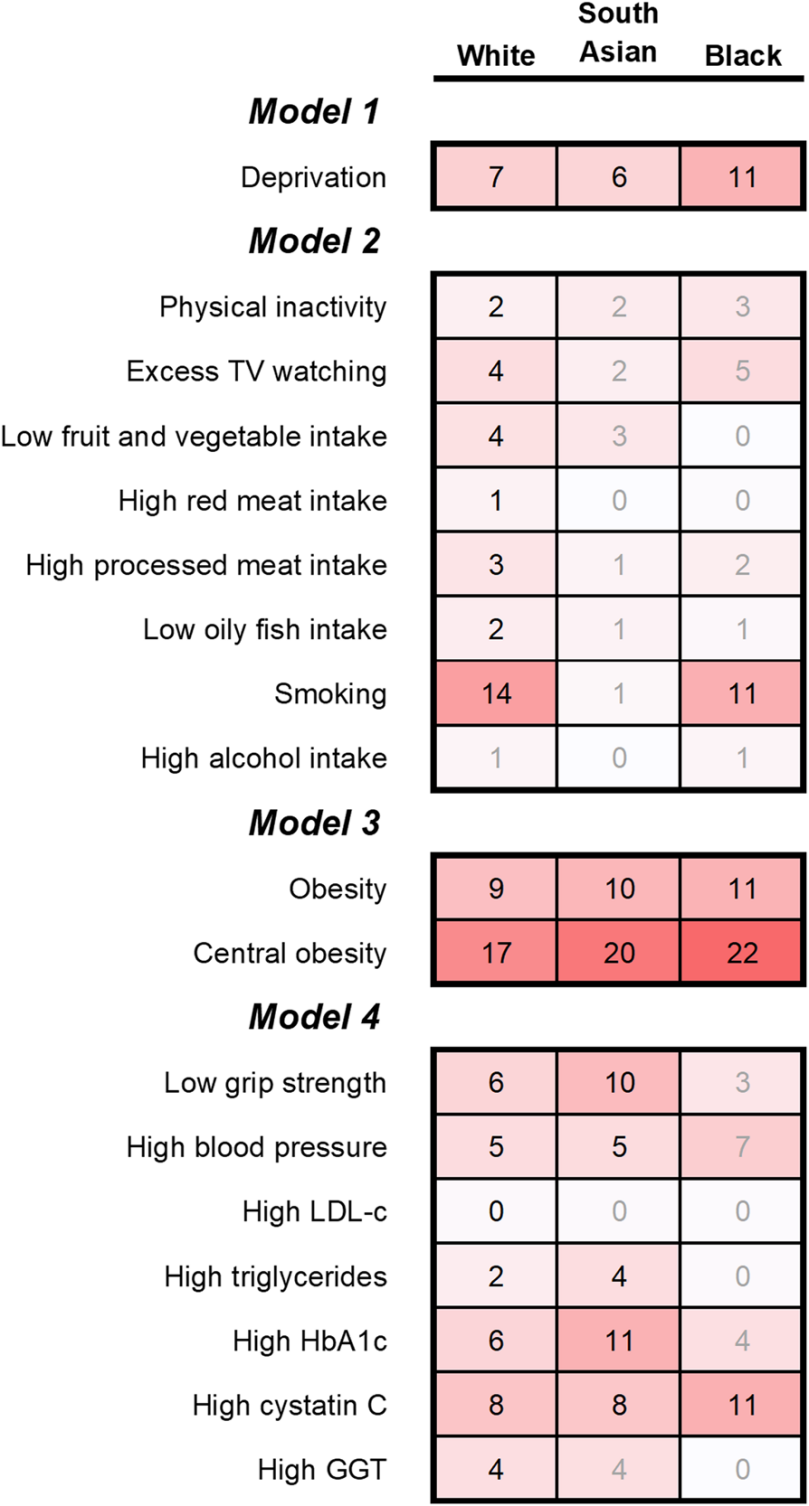
Population attributable fractions for risk factors by ethnic group. Numbers in cells are population attributable fractions for that ethnic group. PAFs with 95% CIs overlapping 0 are greyed out. Model 1: adjusted for age and sex only. Model 2: additionally adjusted for deprivation. Model 3: additionally adjusted for lifestyle factors. Model 4: additionally adjusted for adiposity markers. TV, television; BMI, body mass index; HbA1c, glycated haemoglobin; GGT, gamma-glutamyl transferase. Continuous variables were categorised as quintiles in this analysis. Obesity defined as BMI≥30.0 kg/m^2^, central obesity as WHR >0.85 for women and >0.90 for men, low grip strength as 1st quintile, and high blood pressure, LDL-c, triglycerides, HbA1c, cystatin C, and GGT as the 5th quintile

Based on the HRs shown in Additional file 1: Table S2, the population attributable fractions for the risk factors by ethnic group are shown in Fig. 1 and Additional file 1: Table S3. Adiposity accounted for the highest proportion of CVD cases regardless of ethnicity: 26%, 30%, and 33% among White, South Asian, and Black participants respectively. Smoking made the second largest contribution to CVD cases among White (14%) and Black (11%) participants, following by deprivation in the latter (11%). Among South Asian participants, HbA1c (11%) made the second largest contribution.

## Discussion

### Principal findings

Ethnic differences in CVD risk were due, in part, to differences in the prevalence of risk factors and, in part, to differences in the magnitude of risk associated with these factors. Adiposity was the major contributor to CVD cases in all three groups; however, it contributed to a higher proportion of cases in Black and South Asian participants because of the higher prevalence of obesity and central obesity respectively combined with higher risks associated with obesity. In contrast to White and Black participants, HbA1c was an important contributor in South Asian participants (even when accounted for diabetes diagnosis) and smoking was not. This was due to a higher prevalence of, and a stronger association with, the former and a lower prevalence of, and a weaker association with, the latter. Deprivation was a notable exception to the above risk factors in that the risk associated with deprivation was not different in Black participants, but its prevalence was higher; therefore, it was a large contributor to CVD cases in Black participants and explained all of their excess CVD risk in comparison to White participants. Our data, therefore, suggest that national health care policy and social policy should focus on ethnicity-specific CVD risk factors to reduce the overall burden of CVD in the UK, and improve inequalities in CVD incidence.

### Strengths and weaknesses of this study

Most studies on CVD have excluded some ethnic groups or simply included all ethnic groups in the same model. Our finding of statistical interactions demonstrated that the latter is not appropriate since it assumes that effect sizes are constant across ethnic groups, that is, no differential susceptibility. Atypically, UK Biobank has greater statistical power to test for statistical interactions and undertake sub-group analyses by ethnicity. We took a comprehensive approach to studying ethnic differences in CVD by covering sub-group differences in the prevalence of risk factors (differential exposure), the magnitude of their association with CVD (differential susceptibility), and the resultant population attributable fractions. We considered the causal structure among risk factors to minimise the risk of overadjustment and collider biases. However, this study has several limitations. UK Biobank is not entirely representative of the UK population, with evidence of a healthy volunteer selection bias. Participants who have withdrawn from the UK Biobank might introduce differential misclassification even though the proportion of withdrawal (0.2%) is too small to cause meaningful differences. Estimates of effect size were found to be consistent with more representative cohorts [20], but the prevalence of some risk factors will be lower resulting in underestimates of the PAFs. The calculation of PAFs assumes that factors are causal which may not be true. In spite of adjustment for a wide range of covariates, residual confounding is always possible in observational studies. For less acute CVD such as HF, there might be differential information bias due to differences in care-seeking behaviours by ethnicity. There were varying lengths of follow-up for CV hospitalisation and CV deaths which could result in a higher weighting for fatal (thus more severe) events. There were missing data in some risk factors, e.g. lipid and HbA1c, even though the proportion is quite small (<7%) and should not alter the conclusions. The mediation analysis used in this study is crude and could not accurately estimate the proportion attributable to each of the mediators. However, it should still illustrate the relative importance since the outcome is uncommon (<10%) [15].

### Strengths and weaknesses in relation to other studies

The findings in this study were generally consistent with the literature but meaningfully expand our understanding of CVD risks in underrepresented ethnic groups. Similar to our study, a previous large UK study reported South Asians to be at 29–67% higher risk of CVD when adjustment only included age and sex [8]. However, that study reported lower age- and sex-adjusted risk of myocardial infarction among Black people, whereas we found that their risk only became lower *after* adjusting for deprivation.

Studies have previously shown differences in CVD risk factors by ethnicity [21], but not many have studied how these factors explain the excess risk among ethnic minority groups. A large database linkage study of 1 million people [8] found no meaningful attenuation of associations between South Asian ethnicity and CVD, suggesting no strong mediation. However, their mediator variables only included a subset of those included in the current study. There may also be misclassification bias since the variables were ascertained from primary care data, where CVD risk factors may be more likely to be measured and recorded among people with evidence of CVD. A reanalysis of a trial of 200 participants [22] reported visceral adipose tissue to be a key mediator leading to higher insulin resistance and eventually CVD among South Asians. This is consistent with the current study showing that obesity and central obesity were independently associated with CVD among South Asians, and together accounted for 30% of cases in this group. Of note, other blood measures strongly linked to excess ectopic fat (e.g. triglyceride and HbA1c) were also strongly related to CVD in South Asians.

Previous studies of differential susceptibility by ethnicity have mostly focused on diabetes. Our previous analysis of UK Biobank [6] concluded that South Asians were more susceptible to the elevated risk of diabetes than White people. Another study [23] also found that ethnic differences in CVD mortality were greater among people with diabetes than the general population.

This study estimated that 11% of incident CVD cases in South Asians could be attributed to high HbA1c, slightly higher than a previous estimate of 9% [6]. It should be noted that the previous study only examined physician-diagnosed diabetes. People with HbA1c >38 mmol/mol were found to have slightly higher CVD risk than those at 30–32 mmol/mol and the risk was even higher for those with undiagnosed diabetes [24].

### Meaning of this study

This study showed that adiposity accounts for the highest proportion of CVD composite (including atherosclerotic cardiovascular disease and heart failure) in all three ethnic groups. Population interventions to reduce obesity should benefit all the three ethnic groups and could potentially narrow the ethnic inequalities, since adiposity accounted for higher proportions of CVD in South Asian and Black than White people. This reinforces our recent study showing that adiposity now accounts for more deaths than smoking [25]. It also fits with mounting genetic evidence that supports lifelong excess adiposity as causally linked to multiple CVD outcomes [26]. In addition to adiposity, there may be a benefit in a more targeted approach to other risk factors such as diabetes/impaired glucose tolerance and smoking with a greater focus on ethnic groups at the highest risk. Finally, reductions in socioeconomic inequalities could also reduce ethnic inequalities in CVD, particularly in relation to tackling the higher risk among Black people. This could be particularly relevant given the concerns over ethnic differences in post-COVID-19 recovery [27].

### Unanswered questions and future research

Future research should evaluate whether the effectiveness of strategies to reduce adiposity varies by ethnic groups. Screening for diabetes in the whole UK population has not been supported [28] but future studies are required to determine the cost-effectiveness of screening South Asian communities given their higher prevalence of diabetes and stronger association between diabetes and CVD. The proportion attributable to each of the mediators could be estimated using counterfactual-based analysis using data with repeated measures of mediators.

## Conclusions

Ethnic inequalities in CVD are explained by a combination of differential exposure and differential susceptibility across a range of risk factors. Adiposity is an important contributor to CVD regardless of ethnicity. Ethnic inequalities in CVD may be best tackled by targeting interventions according to ethnic differences in risk profiles. Specifically, the excess risk among South Asians might be tackled by interventions to reduce diabetes and impaired glucose tolerance while interventions to reduce socioeconomic deprivation and smoking could potentially reduce excess risk among Black people.

## Supplementary Information

**Additional file 1: Table S1.** Associations of SBP, lipid, and HbA1c with cardiovascular disease after adjusting for diagnosis of diabetes and medications for blood pressure and cholesterol. **Table S2.** Hazard ratios and 95% CI for PAF analysis. **Table S3.** Population attributable fractions for risk factors by ethnic group.

## Data Availability

Data can be required from the UK Biobank (https://www.ukbiobank.ac.uk/).

## Abbreviations

%RD: Percentage risk reductions
BMI: Body mass index
CI: Confidence interval
CVD: Cardiovascular disease
GGT: Gamma-glutamyltransferase
HbA1c: Glycated haemoglobin
HF: Heart failure
HR: Hazard ratio
IQR: Interquartile range
LDL-c: Low-density lipoprotein cholesterol
MI: Myocardial infarction
PAF: Population attributable fractions
SBP: Systolic blood pressure
SD: Standard deviation
