# Supplementary material for: Ethnic differences in cardiovascular risk: examining differential exposure and susceptibility to risk factors
Source: BMC Med. 2022 Apr 27;20:149. doi: 10.1186/s12916-022-02337-w (PMC9042646; doi:10.1186/s12916-022-02337-w)
Supplement: Supplementary file 1 — Additional file 1: Table S1. Associations of SBP, lipid, and HbA1c with cardiovascular disease after adjusting for diagnosis of diabetes and medications for blood pressure and cholesterol. Table S2. Hazard ratios and 95% CI for PAF analysis. Table S3. Population attributable fractions for risk factors by ethnic group. [file 12916_2022_2337_MOESM1_ESM.docx]

Ethnic differences in cardiovascular risk: examining differential exposure and susceptibility to risk factors

Frederick K Ho^1^, Stuart R Gray^2^, Paul Welsh^2^, Jason M R Gill^2^, Naveed Sattar^2^*, Jill P Pell^1*^, Carlos Celis-Morales^1,2*^

1. Institute of Health and Wellbeing, University of Glasgow, Glasgow, United Kingdom
2. Institute of Cardiovascular & Medical Sciences, Glasgow, United Kingdom

Corresponding author:

Frederick K Ho

[Frederick.Ho@glasgow.ac.uk](mailto:Frederick.Ho@glasgow.ac.uk)

1 Lilybank Gardens

Glasgow, G12 8RZ

United Kingdom

Additional File 1

**Table S1**. Associations of SBP, lipid, and HbA1c with cardiovascular disease after adjusting for diagnosis of diabetes and medications for blood pressure and cholesterol.

|  | **White** | **South Asian** | **Black** |
| --- | --- | --- | --- |
|  | **HR (95% CI)** | **HR (95% CI)** | **HR (95% CI)** |
| SBP | **1.14 (1.12-1.15)** | **1.18 (1.10-1.27)** | **1.11 (1.01-1.22)** |
| LDL-c | **1.10 (1.09-1.12)** | **1.17 (1.08-1.27)** | 1.08 (0.97-1.19) |
| Triglycerides | **1.05 (1.04-1.06)** | **1.12 (1.05-1.18)** | 1.10 (0.97-1.24) |
| HbA1c | **1.09 (1.08-1.11)** | **1.12 (1.07-1.18)** | **1.05 (0.97-1.14)** |

HR: hazard ratio; CI confidence interval; LDL-c: low-density lipoprotein cholesterol; HbA1c: glycated haemoglobin; GGT: gamma-glutamyl transferase

Adjusted for age and sex, deprivation, lifestyle factors, adiposity markers, diagnosis of diabetes and medications for blood pressure and cholesterol

**Table S2**. Hazard ratios and 95% CI for PAF analysis.

|  | **White** | **South Asian** | **Black** |
| --- | --- | --- | --- |
| **Model 1** |  |  |  |
| Deprivation | 1.17 (1.03-1.32) | 1.25 (1.04-1.50) | 1.40 (1.37-1.43) |
| **Model 2** |  |  |  |
| Physical inactivity | 1.12 (0.97-1.30) | 1.25 (1.01-1.55) | 1.07 (1.04-1.10) |
| Excess TV watching | 1.21 (0.99-1.47) | 1.19 (0.96-1.49) | 1.26 (1.22-1.29) |
| Low fruit and vegetable intake | 1.20 (1.01-1.42) | 0.78 (0.61-1.01) | 1.14 (1.11-1.17) |
| High red meat intake | 1.09 (0.89-1.32) | 0.97 (0.78-1.20) | 1.05 (1.02-1.08) |
| High processed meat intake | 1.05 (0.88-1.25) | 1.22 (0.98-1.52) | 1.07 (1.04-1.09) |
| Low oily fish intake | 1.16 (1.01-1.34) | 1.08 (0.72-1.64) | 1.20 (1.16-1.24) |
| Current smoking | 0.94 (0.78-1.14) | 1.52 (1.21-1.91) | 1.20 (1.17-1.23) |
| Former smoking | 1.10 (0.89-1.37) | 1.68 (1.28-2.22) | 1.87 (1.81-1.93) |
| High alcohol intake | 1.04 (0.81-1.34) | 1.05 (0.74-1.49) | 0.99 (0.96-1.02) |
| **Model 3** |  |  |  |
| Obesity | 1.64 (1.41-1.90) | 1.25 (1.03-1.52) | 1.37 (1.34-1.40) |
| Central obesity | 1.31 (1.11-1.54) | 1.52 (1.23-1.87) | 1.29 (1.26-1.32) |
| **Model 4** |  |  |  |
| Low grip strength (1^st^ quintile) | 1.26 (1.08-1.46) | 1.16 (0.90-1.48) | 1.28 (1.25-1.31) |
| High blood pressure (5^th^ quintile) | 1.29 (1.08-1.54) | 1.29 (1.01-1.65) | 1.27 (1.24-1.30) |
| High LDL-c (5^th^ quintile) | 1.10 (0.90-1.35) | 0.88 (0.61-1.26) | 1.08 (1.05-1.11) |
| High triglycerides (5^th^ quintile) | 1.27 (1.08-1.48) | 0.91 (0.62-1.34) | 1.08 (1.05-1.11) |
| High HbA1c (5^th^ quintile) | 1.32 (1.13-1.54) | 1.12 (0.90-1.41) | 1.24 (1.21-1.28) |
| High cystatin C (5^th^ quintile) | 1.27 (1.09-1.49) | 1.88 (1.46-2.42) | 1.31 (1.28-1.35) |
| High GGT (5^th^ quintile) | 1.11 (0.93-1.33) | 0.94 (0.73-1.20) | 1.14 (1.11-1.17) |

Model 1: adjusted for age and sex only

Model 2: adjusted for deprivation additionally

Model 3: adjusted for lifestyle factors additionally

Model 4: adjusted for adiposity markers additionally

TV: television; BMI: body mass index; HbA1c: glycated haemoglobin; GGT: gamma-glutamyl transferase

Obesity defined as BMI≥30.0 kg/m^2^; central obesity as WHR >0.85 for women and >0.90 for men; low grip strength as 1^st^ quintile; high blood pressure, LDL-c, triglycerides, HbA1c, cystatin C, and GGT as the 5^th^ quintile.

**Table S3**. Population attributable fractions for risk factors by ethnic group.

|  | **White** | **South Asian** | **Black** |
| --- | --- | --- | --- |
| **Model 1** |  |  |  |
| Deprivation | 6.52 (6.09-6.96) | 5.74 (1.86-9.61) | 10.92 (1.60-20.25) |
| **Model 2** |  |  |  |
| Physical inactivity | 1.73 (1.31-2.15) | 2.37 (-0.33-5.07) | 2.98 (-0.58-6.55) |
| Excess TV watching | 4.46 (4.07-4.84) | 1.87 (0.08-3.66) | 4.59 (0.84-8.34) |
| Low fruit and vegetable intake | 3.93 (3.49-4.36) | 2.94 (0.68-5.20) | -0.23 (-3.87-3.41) |
| High red meat intake | 1.35 (0.99-1.72) | -0.32 (-2.13-1.48) | -2.41 (-6.51-1.69) |
| High processed meat intake | 3.34 (2.71-3.97) | 1.08 (-1.38-3.54) | 1.96 (-1.98-5.90) |
| Low oily fish intake | 2.13 (1.83-2.43) | 1.25 (-2.02-4.51) | 0.58 (-1.09-2.26) |
| Smoking | 13.98 (13.14-14.82) | 0.59 (-2.36-3.54) | 11.45 (6.45-16.46) |
| High alcohol intake | 0.94 (0.44-1.45) | -0.32 (-1.71-1.07) | 0.91 (-1.11-2.94) |
| **Model 3** |  |  |  |
| Obesity | 9.06 (8.51-9.61) | 10.33 (7.36-13.29) | 10.92 (4.32-17.51) |
| Central obesity | 16.97 (15.89-18.06) | 19.92 (11.62-28.21) | 22.08 (13.12-31.04) |
| **Model 4** |  |  |  |
| Low grip strength (1^st^ quintile) | 5.77 (5.23-6.30) | 9.62 (4.03-15.21) | 3.19 (-1.46-7.84) |
| High blood pressure (5^th^ quintile) | 4.58 (4.01-5.16) | 4.68 (1.84-7.52) | 6.81 (1.93-11.69) |
| High LDL-c (5^th^ quintile) | 0.40 (-0.08-0.88) | 0.16 (-1.96-2.27) | -0.63 (-3.47-2.20) |
| High triglycerides (5^th^ quintile) | 2.19 (1.63-2.74) | 4.41 (0.74-8.07) | 0.14 (-2.32-2.59) |
| High HbA1c (5^th^ quintile) | 5.70 (5.13-6.28) | 11.04 (4.80-17.28) | 4.17 (-5.96-14.30) |
| High cystatin C (5^th^ quintile) | 8.20 (7.53-8.86) | 7.88 (2.97-12.79) | 11.38 (6.71-16.04) |
| High GGT (5^th^ quintile) | 4.27 (3.69-4.84) | 4.22 (1.21-7.23) | -0.30 (-5.51-4.91) |

Model 1: adjusted for age and sex only

Model 2: adjusted for deprivation additionally

Model 3: adjusted for lifestyle factors additionally

Model 4: adjusted for adiposity markers additionally

TV: television; BMI: body mass index; HbA1c: glycated haemoglobin; GGT: gamma-glutamyl transferase

Obesity defined as BMI≥30.0 kg/m^2^; central obesity as WHR >0.85 for women and >0.90 for men; low grip strength as 1^st^ quintile; high blood pressure, LDL-c, triglycerides, HbA1c, cystatin C, and GGT as the 5^th^ quintile.
